# Intravitreal Polymeric Nanocarriers with Long Ocular Retention and Targeted Delivery to the Retina and Optic Nerve Head Region

**DOI:** 10.3390/pharmaceutics13040445

**Published:** 2021-03-26

**Authors:** Vijayabhaskarreddy Junnuthula, Amir Sadeghi Boroujeni, Shoupeng Cao, Shirin Tavakoli, Roxane Ridolfo, Elisa Toropainen, Marika Ruponen, Jan C. M. van Hest, Arto Urtti

**Affiliations:** 1 Drug Research Program, Faculty of Pharmacy, University of Helsinki, Viikinkaari 5 E, 00790 Helsinki, Finland; junnuthula.vijayabhaskarreddy@helsinki.fi (V.J.); shirin.tavakoli@helsinki.fi (S.T.); 2 School of Pharmacy, University of Eastern Finland, Yliopistonranta 1, 70211 Kuopio, Finland; amir.sadeghi@uef.fi (A.S.B.); elisa.toropainen@uef.fi (E.T.); marika.ruponen@uef.fi (M.R.); 3 Bio-Organic Chemistry, Eindhoven University of Technology, P.O. Box 513 (STO 3.31), 5600 MB Eindhoven, The Netherlands; caos@mpip-mainz.mpg.de (S.C.); roxane.ridolfo@laposte.net (R.R.); J.C.M.v.Hest@tue.nl (J.C.M.v.H.); 4 Laboratory of Biohybrid Technologies, Institute of Chemistry, St. Petersburg State University, Peterhof, 198504 St. Petersburg, Russia

**Keywords:** intravitreal, polymersome, polymeric micelle, drug delivery, retina, optic nerve

## Abstract

Posterior eye tissues, such as retina, are affected in many serious eye diseases, but drug delivery to these targets is challenging due to various anatomical eye barriers. Intravitreal injections are widely used, but the intervals between invasive injections should be prolonged. We synthesized and characterized (^1^H NMR, gel permeation chromatography) block copolymers of poly(ethylene glycol), poly(caprolactone), and trimethylene carbonate. These polymers self-assembled to polymersomes and polymeric micelles. The mean diameters of polymersomes and polymeric micelles, about 100 nm and 30–50 nm, respectively, were obtained with dynamic light scattering. Based on single particle tracking and asymmetric flow field-flow fractionation, the polymeric micelles and polymersomes were stable and diffusible in the vitreous. The materials did not show cellular toxicity in cultured human umbilical vein endothelial cells in the Alamar Blue Assay. Pharmacokinetics of the intravitreal nanocarriers in the rabbits were evaluated using in vivo fluorophotometry. The half-lives of the polymersomes (100 nm) and the micelles (30 nm) were 11.4–32.7 days and 4.3–9.5 days. The intravitreal clearance values were 1.7–8.7 µL/h and 3.6–5.4 µL/h for polymersomes and polymeric micelles, respectively. Apparent volumes of distribution of the particles in the rabbit vitreous were 0.6–1.3 mL for polymeric micelles and 1.9–3.4 mL for polymersomes. Polymersomes were found in the vitreous for at least 92 days post-dosing. Furthermore, fundus imaging revealed that the polymersomes accumulated near the optic nerve and retained there even at 111 days post-injection. Polymersomes represent a promising technology for controlled and site-specific drug delivery in the posterior eye segment.

## 1. Introduction

Many vision impairing diseases affect the tissues of the posterior eye segment, such as retina, choroid, and optic nerve [1]. For example, age-related macular degeneration, diabetic retinopathy, macular edema, and glaucoma cause visual impairment in millions of patients due to the impaired functionality of the retina and the optic nerve.

Drug delivery to these posterior eye tissues is challenging due to the presence of various anatomical eye barriers [2]. Topical ocular drug administration is preferred, but it is effective only in the treatment of anterior eye segment diseases [3] and, as a consequence, intravitreal injection is the method of choice in drug administration to the posterior eye segment [2]. For example, about 22 million anti-VEGF injections are given annually worldwide to treat wet form of age-related macular degeneration, and the number is continuously increasing [4]. Intravitreal injection delivers the entire drug dose to the vitreous, but this procedure requires skill, causes discomfort to the patient, and involves burdensome logistics for patients and healthcare at high numbers of current injections. For instance, intravitreal injections of corticosteroid suspensions and solutions of anti-VEGF proteins are used in the clinics at intervals of a few weeks to two months in chronic treatment for years or even for the entire lifetime [5]. Therefore, prolongation of the injection intervals is desirable. Currently, corticosteroid implants are available for intravitreal drug delivery, but improved controlled drug delivery technologies are needed to allow injections at long time intervals and targeted delivery into the retina and the optic nerve regions [2,5].

Nanoparticles for drug delivery can be prepared in various forms, including polymeric nanoparticles, micelles, liposomes, dendrimers, polymersomes, and polymeric conjugates [6]. Polymersomes are versatile nanoparticles that are made of di- or tri block copolymers that self-assemble to form polymeric bilayer walls around the aqueous core [7,8,9]. Polymeric micelles are also formed in a self-assembly process, but in this case, a core–shell structure is formed with a non-polar inner domain and a polar outer layer [10]. Because of their size and reservoir function, they could be useful to prolong the presence of therapeutic agents in the vitreous and not impair vision, which could be an issue for micron-sized particles. Advantages of polymersomes as compared to other nanocarriers include (1) lack of harsh manufacturing conditions (e.g., high temperatures, high-energy processing); (2) encapsulation of both hydrophilic and lipophilic drugs; (3) versatile options in terms of particle size, charge, rigidity, shape, and drug release [8,10]. Even though some literature about ocular nanoparticle delivery is available [11], distribution and elimination of these systems is poorly understood because indirect or qualitative analytical methods have been used [2]. Polymersomes have not been tested as intravitreal drug delivery systems, whereas polymeric micelles showed intravitreal half-lives of 2–5 days in rabbits [12,13,14].

We tested polymersomes and polymeric micelles as potential intravitreal drug delivery systems. Amphiphilic block copolymers based on poly(ethylene glycol) (PEG), poly(caprolactone) (PCL), and trimethylene carbonate (TMC) were synthesized. These polymers were chosen based on physico-chemical advantages (e.g., reliable formation of polymeric micelles and polymersomes) and their general biocompatibility. The block copolymers self-assembled to polymeric micelles and polymersomes that were tested in terms of physicochemical properties, interactions with vitreous fluid, and ocular kinetics in rabbits after intravitreal injections. The polymersomes showed extraordinarily long ocular retention and targeted disposition to the retina and the optic nerve head regions.

## 2. Materials

All the starting materials were purchased from commercial suppliers. The materials, including trimethylene carbonate (Actu-All Chemicals), and Ɛ-caprolactone (Fluorochem UK), *N*,*N’*-dicyclohexylcarbodiimide (DCC) (Acros Organics), and BODIPY (Borondipyrromethene) -FL carboxylic acid (Lumiprobe) were used as received. All other chemicals obtained from Thermo Scientific (Waltham, MA, USA) and Merck Inc. (London, UK).

## 3. Methods

### 3.1. Synthesis and Characterization of Block Copolymers

Synthesis protocols are summarized in the supplementary scheme (Supplementary Material Section 1).

**Micelles.** The block copolymer synthesis of poly(ethylene glycol)-b-poly(trimethylene carbonate) (p22, mPEG_22_-PTMC_38_) and p42 (mPEG_42_-PTMC_38_) was the same as reported earlier [15]. The critical micelle concentrations were determined as described earlier [15].

**Polymersomes.** The synthesis of poly(ethyleneglycol)-b-poly(ε-caprolactone)-g-poly(trimethylenecarbonate) (PEG_22-_PCl_30_gTMC_30_) (PPP) block copolymers has been previously reported [16]. The synthesis schemes, including fluorescently labeled block copolymers, are in the Supplementary Materials (Supplement part A). Synthesis products were identified with NMR. The ^1^H NMR spectra were recorded on a Bruker Advance 400 MHz spectrometer with CDCl_3_ as a solvent and tetramethylene silane (TMS) as internal standard (Supplementary Material Section 1).

Gel permeation chromatography (GPC) was used for molecular weight estimation. Shimadzu Prominence GPC system was used with a PLgel MIXED D column (5 µm; Polymer Laboratories) with a differential refractive index detector. Tetrahydrofuran (THF) was used as an eluent at a flow rate of 1 mL/min.

### 3.2. Preparation and Physical Properties of Self-Assembling Nanoparticles

**Micelles**. PEG-PTMC blank micelles were formulated by dissolution of the copolymers in 10% oligo(ethylene glycol), MW = 350 (PEG350) in a 5 mL vial at 50 °C. Mixing at 250 rpm was used to stir the copolymer solution that was then subsequently hydrated with 1 mL of water.

**Polymersomes**. Volume of 20 μL of PEG−b-p(CL-g-TMC) block copolymer (PPP) in 10% in PEG 350 (*w/w*) was pipetted into a 5 mL vial. The temperature was kept at 50 °C. A thin film of the polymer solution was generated by magnetic stirring at 250 rpm. Subsequently, the film was hydrated with 80 μL of water followed by continuous stirring for 5 min. Polymersomes were then diluted until the desired concentrations.

**Fluorescently labeled polymersomes and micelles.** Dye loaded polymersomes were prepared in the same manner as non-labeled polymersomes, but 10% of the PPP polymer was replaced with dye conjugated polymer (BPPP) (labeled/unlabeled ratio = 1/9). Likewise, dye loaded micelles were prepared by replacing 10% of the non-labeled polymer with the labeled one (ratio labeled/non-labeled = 1:9). The thin film hydration method remained the same.

**Size and zeta-potential.** Dynamic light scattering (DLS) was used for the size measurements of the polymeric particles. The measurements were conducted with a Malvern APS Zetasizer (Malvern Instruments, Westborough, MA, USA). An average of 10 number intensity readings from three samples were reported. The measurement buffer was 10 mM phosphate buffered saline (PBS), pH 7.4. All solutions were made in 18.2 MΩ deionized water purified through the Milli-Q water purification system (Millipore Corporation, Burlington, MA, USA). The samples were measured in DTS1070 sample cells (zeta potential measurement) or in 96-well plates (particle size measurement) (Nunc, Thermofisher Inc., Waltham, MA, USA).

**Particle stability in vitreous**. Vitreous humor was isolated from porcine eyes [17,18] that were obtained from the local slaughterhouse in Finland and was used as the matrix to study stability of the polymeric micelles and polymersomes. Briefly, the porcine vitreous was mixed with polymersomes and polymer micelles at ratio of 1:1 and incubated at 37 °C. The samples at various time points were analyzed for hydrodynamic radius using asymmetric flow field-flow fractionation, multi-angle light scattering, and quasi elastic light scattering (AF4-MALS-QELS).

AF4-MALS-QELS experiments were performed on a Wyatt Eclipse AF4 instrument connected to a Shimadzu LC-20A Prominence system with Shimadzu CTO20A injector. The AF4 was further connected to the following detectors: a Shimadzu SPD20A UV detector; a Wyatt DAWN HELEOS II light scattering detector (MALS) installed at different angles (12.9°, 20.6°, 29.6°, 37.4°, 44.8°, 53.0°, 61.1°, 70.1°, 80.1°, 90.0°, 99.9°, 109.9°, 120.1°, 130.5°, 149.1°, and 157.8°) using a laser operating at 664.5 nm; a QELS detector installed at an angle of 140.1° and a Wyatt Optilab Rex refractive index detector. The detectors were calibrated with bovine serum albumin. The processing and the analysis of the light scattering data and the radius of gyration calculations were performed on Astra 6.1.1 software. Berry model for particle size > 50 nm was used. All AF4 fractionations were performed on an AF4 short channel with regenerated cellulose membrane (10 kDa; Millipore) and spacer of 350 μm. Flow parameters for fractionation are described in the Supplementary Materials (Tables S1 and S2).

**Diffusion in the vitreous.** Vitreal diffusion of polymeric nanoparticles was studied in porcine eyes as described earlier [19]. Briefly, fresh porcine eyes were obtained from a slaughterhouse (HKScan, Forssa, Finland). The extraocular connective tissues such as muscles were removed with a surgical scissor. The eyes were subsequently dipped in 70% ethanol and kept in PBS at +4 °C. The anterior part of the eye was gently cut and removed to make the intact vitreous ready for the injection. Then, 50 µL (0.25 mg/mL) of fluorescently labeled polymersomes were injected into the vitreous of each eyecup at a depth of 0.5 cm with 30 G insulin syringe (BD, Franklin Lakes, NJ, USA). A microwell dish with a glass window (MatTek Corporation, Ashland, MA, USA) was gently placed on the cut surface and flipped over, leaving the cut vitreous facing down. The visualization procedure was performed with confocal microscopy (Marianas 3i, Intelligent Imaging Innovation Inc. Denver, CO, USA) using Slidebook^®^Software V.6 (Intelligent Imaging Innovation Inc., Denver, CO, USA). Vitreal movements were analyzed by @msdanalyzer MATLAB plugin of Imaris 9.3.1 software (Bitplane AG, Zurich, Switzerland) as we previously reported [19]. Finally, diffusion coefficient in vitreous (D_v_) was calculated from mean square displacement (MSD) versus time plot using the following equation:

D_v_ = MSD/4τ (1)

where MSD of tracks are computed in a two-dimensional space, and τ is the time delay for the calculated movement.

### 3.3. In Vivo Experiments in Rabbits 

**Endotoxin tests.** Polymeric nanoparticle preparations were tested for endotoxins by utilizing Pierce LAL Chromogenic Endotoxin Quantification Kit (Thermo Scientific).

**Particle kinetics.** In vivo pharmacokinetic measurements were performed using fluorophotometer (Fluorotron, OcuMetrics, Mountain view, CA, USA). At first, the correlation between the fluorescent signal in labeled polymersomes and polymeric micelles and their concentrations was evaluated. Linear standard curve with high correlation coefficient (r^2^ = 0.987) was used for particle quantification in the eye. Dutch belted pigmented rabbits were anesthetized with subcutaneous injection of ketamine (dose 25 mg/kg) and medetomidine (dose 0.5 mg/kg) (Domitor vet 1 mg/mL; Orion Pharma, Espoo, Finland). The eyes were dilated with one drop of topical tropicamide (Oftan Tropicamid 5 mg/mL, Santen Pharmaceutical Co, Osaka, Japan). The eyes were locally anesthetized with one drop of oxybuprocaine (Oftan Obucain 4 mg/mL, Santen Pharmaceutical Co., Ltd., Tampere, Finland) just before intravitreal injection. The autofluorescence of the eyes was measured before intravitreal injection of labeled polymersomes (dose 320 µg) and polymeric micelles (dose 500 µg). The needle size for intravitreal injection was 30 G, and the volume of injection was 50 µL per eye. The fluorophotometry scans were performed at different time points for about 3.5 months. During scanning, the rabbits were under sedation by subcutaneous injection of Domitor (Domitor vet 1 mg/mL; 0.3 mL/kg). Nanocarrier levels were quantitated in the vitreous using the average concentration in the middle of the vitreous (starting at 3 mm of relative distance from retina and ending at 13 mm from retina). The results were further used to derive pharmacokinetic parameters of the polymersomes and micelles in the vitreous using Phoenix Software (version 6.3, Pharsight Inc., St. Louis, MO, USA).

**Fundus imaging**. The pupil was dilated using topical tropicamide (one drop of Oftan Tropicamid 5 mg/mL) in each eye 15 min before doing scans, and the fundus images were captured by Micron IV Retinal Imaging Microscope (Phoenix Technology Group, Pleasanton, CA, USA) using full color and green fluorescence sets of filters. The rabbits were under anesthesia using ketamine (dose 25/kg) and medetomidine (dose 0.5 mg/kg) cocktail during the imaging.

**Retinal microscopic imaging**. At the end of the study, the animals in the polymersome group were sacrificed, and the eyes were enucleated. The eyes were fixed in Davidson solution for 48 h. Thereafter, they were kept in PBS at 4 °C. Before microscope imaging, the anterior tissues and part of the vitreous were removed. The images collected are from the surface of retina (Leica Stereomicroscope with fluorescence Immuno Diagnostic Ltd., LMS, Espoo, Finland).

**Animal study permit.** All animal experiments were approved according to the ethical guidelines at University of Eastern Finland (license number ESAVI/8621/04.10.07/2017, 13 December 2017). All the experiments were carried out by authorized personnel.

## 4. Results

### 4.1. Characterization of Polymeric Micelles and Polymersomes

The block copolymers (PEG-PCL-TMC) were optimized to obtain polymersomes with different surface charges (cationic, anionic, and neutral). PEG was used as a hydrophilic block, whereas the gradient block of PCL and PTMC acted as a hydrophobic domain (Figure S1). For the positively charged polymersomes, the hydrophilic end of the PEG block was modified with primary amines end to yield positive surface charge, while carboxylic acid end groups were used for negative charge (Figures S2 and S3). The PEG chain without modification bears almost neutral charge. The dye BODIPY was conjugated to the end of the hydrophobic segment, thereby placing the dye into the bilayer of the polymersomes (Figure S4). The micelles were composed of PEG-PTMC. In that case, no additional surface charge was incorporated. The mean molecular weights (M_n_*)* of all PPP copolymers for polymersomes were in the range of 10.4–14.4 kDa, and polydispersities were 1.13–1.23. The properties of PEG-PTMC polymers for polymeric micelles were described previously [20].

The thin film hydration method produced polymersomes with diameters in the range of ≈ 100 nm (Table 1). Modifications of block copolymers resulted in different zeta potentials (neutral, positive, negative). In the case of polymeric micelles, the sizes were approximately 30–50 nm, and zeta potential values were close to neutrality.

### 4.2. Polymersome Interactions with Ex Vivo Vitreous Humour 

**Figure 1:**
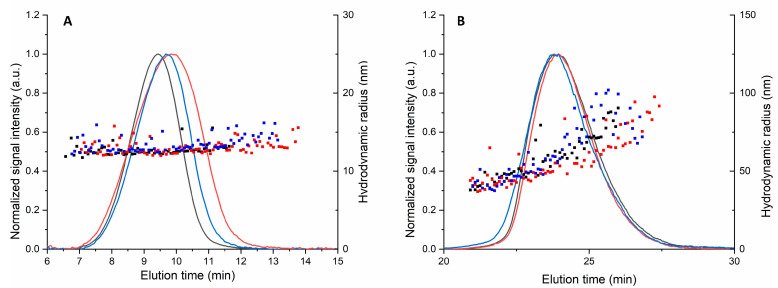
Asymmetric flow field-flow fractionation (AF4)-dynamic light scattering (DLS) data for (**A**) polymeric micelles (p22) and (**B**) polymersomes. Scattered dots indicate the hydrodynamic diameters, and the lines show normalized signal intensities at 280 nm. The measurements were done at 37 °C in porcine vitreous immediately after exposure (black) and after incubation of one day (red) and seven days (blue).

**Stability**. Stability of selected polymeric micelles (p22) and polymersomes (PPP) was studied in porcine vitreous. The size of polymeric micelles remained unchanged, whereas the size of polymersomes increased. However, the particles remained completely separated without aggregation in the AF4 experiments (Figure 1). The elution times of polymersomes were similar before and after exposure to the biological matrix.

**Diffusion.** The vitreal mobility of polymersomes was studied in intact porcine vitreous. Diffusion of the particles in vitreous (D_v_), determined by single particle tracking, indicates that the particles had only small differences in their mobility (Table 2). In general, the polymeric particles were 15–18 times less mobile compared to their theoretical diffusivity in water (D_w_). Apparently, the positive charge (pPPP) was not high enough to significantly hinder diffusion. According to the D_w_/D_v_ values of 15–18, the vitreous does not pose a major barrier for polymersome mobility in the vitreous. Polymeric micelles were too small to be studied with single particle tracking method.

### 4.3. Particle Kinetics In Vivo after Intravitreal Injection 

**Material safety**. Before in vivo experiments in rabbits, we tested cellular toxicity of the polymeric materials in cultured HUVEC cells (Human Umbilical Vein Endothelial Cells) (Supplementary Material Section 3). The particles were safe in the cell experiments even at 50 mg/mL concentration (Figure S5).

Endotoxin levels were at acceptable levels for intravitreal medications. The endotoxin concentrations in the formulations were below the limit of detection of the test (0.1 EU/mL). This means that the amount of endotoxins was less than 0.005 EU in the injected dose (50 µL). This is clinically acceptable, as the limit set by the U.S. Food and Drug Administration (FDA) is 0.2 EU/injection for intraocular devices.

No adverse effects were seen in the eyes after intravitreal injections of the polymeric micelles and polymersomes.

**Figure 2:**
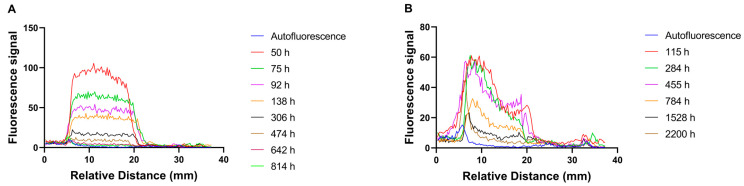
Example fluorophotometer scans of rabbit eyes after injections of (**A**) polymer micelles and (**B**) polymersomes into the rabbit vitreous. Relative distance at about 7–18 mm shows the fluorescence levels in the rabbit vitreous. Autofluorescence is shown as blue line. The other lines indicate the fluorescence levels at different times post-injection.

**Vitreal kinetics**. Fluorescently labeled selected polymeric formulations (p22, PPP) exhibited linear fluorescence over wide concentration range (Figure S6). In vivo fluorophotometry enabled non-invasive monitoring of polymeric micelles and polymersomes in the vitreous humor of the rabbits (Figure 2 and Figure S7). No signal could be detected in the control eyes.

**Figure 3:**
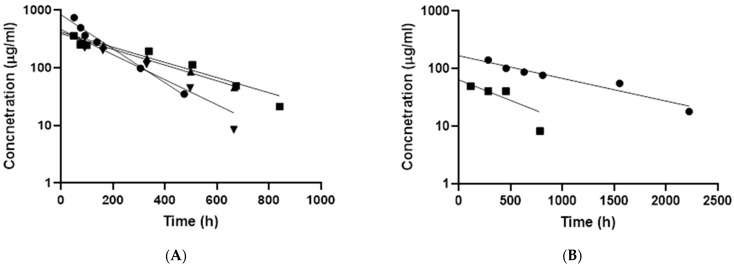
(**A**) Kinetic profiles of polymeric micelles (*n* = 4) and (**B**) polymersomes (*n* = 2) in rabbit vitreous after intravitreal injections. The dots show the experimental data, and lines are the best fits with one-compartmental model with first-order elimination kinetics.

The one compartment model was used to calculate kinetic parameters for intravitreally injected polymeric particles in rabbits (Figure 3, Table 3). Half-lives of polymeric micelles (p22) in the rabbit vitreous were 4–9 days, whereas the half-lives of polymersomes (PPP) were 11–33 days. The volumes of particle distribution in the vitreal cavity (0.6–3.4 mL) were in the same range with the anatomical volume of rabbit vitreous (1.6 mL) (Table 3). Interestingly, axial concentration gradient was seen in the vitreous with polymersomes but not with polymeric micelles (Figure 2 and Figure S7). Polymersome concentrations were highest in the posterior segment of the vitreous, close to the retina (Figure 2 and Figure S7).

**Figure 4:**
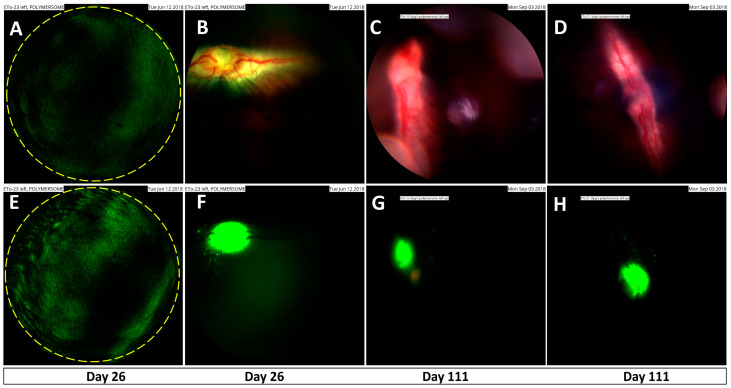
Fundus imaging in rabbit eyes after intravitreal injection of BODIPY labeled neutral polymersomes (PPP). Upper panel shows full color imaging and lower panel represents fluorescence imaging. (**A**,**B**) Polymersome distribution in the rabbit vitreous after 26 days; (**C**–**F**) accumulation of polymersomes at the optic nerve head region; (**G**,**H**) accumulation of polymersomes at the optic nerve head region (horizontal axis). Without injection fluorescence images would be completely black.

**Distribution to the retina and optic nerve region.** Distribution of the polymersomes in the posterior eye segment was also studied with fundus imaging for 111 days post-injection. Green fluorescence of the polymersomes particles showed a uniform particle distribution in the vitreous (Figure 4A,B). The polymersomes accumulated at the optic nerve area after 26 days (Figure 4C,D), and the accumulation at the optic nerve head was still evident at 111 days post-injection (Figure 4E–H).

**Figure 5:**
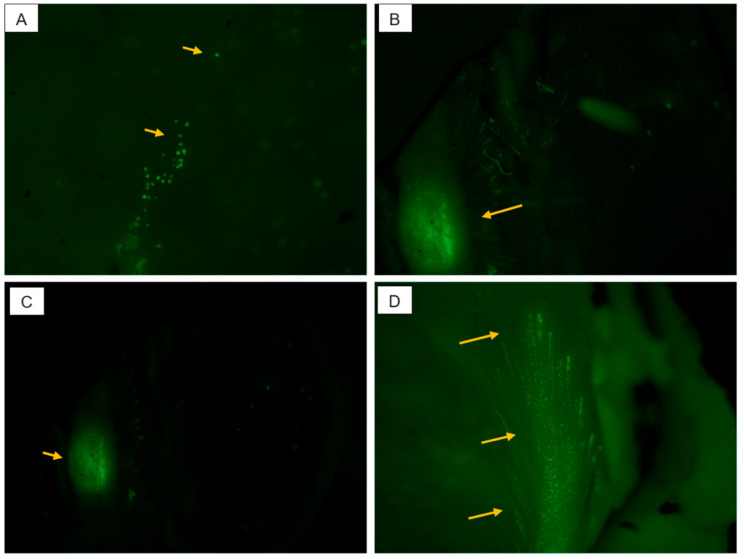
Micrographs (20×) of frozen rabbit vitreous (**A**), optic nerve polymersome accumulation (**B**,**C**) and polymersomes at retinal tissue layers (**D**). The arrows show localization of the polymersomes.

Micrographs from the fixed rabbit vitreous after intravitreal injections are shown in Figure 5. These figures show the presence of neutral polymersomes in the retinal layers and in the optic nerve region of the rabbits.

## 5. Discussion

Current clinical product for intravitreal injections do not include any nanoparticle systems. The products are injectable drug solutions (e.g, antibiotics, anti-VEGF biologicals), coarse drug suspensions (e.g., poorly water-soluble corticosteroids), and implants (e.g., corticosteroids). In principle, nanoparticles would allow use of minimal needle size and prolonged drug action.

We demonstrate polymersome retention of at least 92 days in the rabbit vitreous after intravitreal injection, whereas the polymeric micelles retained for about one month (Figure 3). Long retention of polymersomes in the vitreous is remarkable compared to other nanosized particles. Previously, liposomes showed durations of days [21,22,23,24,25] and albuminated polylactide particles up to two months [26]. Long retention of polystyrene particles was demonstrated in one study, but polystyrene is not a feasible material for drug delivery [27]. Our finding is important for three reasons: (1) long retention enables prolonged local drug delivery—an important goal, since prolonged injection intervals are needed in the clinics; (2) long vitreal residence increases chances of retinal permeation of the drug delivery particles; (3) polymersomes were well tolerated showing no adverse effects in the rabbit eyes. Indeed, we showed localization of polymersomes to the retinal and the optic nerve regions of the rabbit eyes, opening possibilities for targeted drug delivery. Localization of polymersomes at the optic nerve head may have therapeutic implications, since optic nerve is degenerated in glaucoma [28]. No optic nerve reviving treatments are available at the moment. As polymersomes show appealing prospects in drug delivery to the retina and the optic nerve, more detailed studies on their disposition are warranted. Our recent ex vivo study demonstrated retinal permeation of small liposomes and tubular polymersomes [20,29]. Small particle size and non-viscous polymersome formulations enable use of small injection needles (in this study 30 G), representing another clinical advantage compared to the implants.

Elimination mechanisms of nanoparticles from the vitreal cavity are not known, but it is likely that they are eliminated anteriorly, such as intravitreal biologicals [2,30]. This mechanism involves diffusion in the vitreous to the aqueous humor and further elimination in outflow via trabecular meshwork. Anterior elimination should be controlled by the diffusivity in the vitreous humor [2]. Therefore, our results are logical—small micelles are eliminated faster than the larger polymersomes. On the other hand, vitreal diffusivity of polymersomes is similar to the mobility of liposomes [19], but the polymersomes showed much slower elimination in vivo. It is important to note that vitreal fluid convection may also play a role in particle distribution, and some biological mechanisms may be involved, possibly mediated by the protein corona formation in the vitreous [19]. The role of convection is supported by the differences in the vitreal distribution of polymersomes and polymeric micelles. Distribution of larger polymersomes is expected to be more dependent on convection than the distribution of smaller polymer micelles, leading to high particle levels at the posterior part of the vitreous as compared to the levels in the anterior vitreous (Figure 2 and Figure S7). This is in line with fundus and microscopic images which show accumulation of polymersomes in the retina and the optic nerve at terminal timepoints (Figure 4 and Figure 5). Neither intravitreal concentration gradients nor accumulation in the optic nerve were seen in the micelle data (Figure 2 and Figure S7).

Even though protein concentration in the vitreous is much less than in plasma, protein corona is formed on the liposomes upon vitreal contact, as we showed using a workflow that involves surface plasmon resonance and mass spectrometry [19,31]. Herein, we applied AF4 for the first time to investigate nanoparticle aggregation in the vitreous. Importantly, no aggregation of polymeric micelles or polymersomes was observed. Lack of aggregation is essential for retinal targeting, since aggregates are not able to diffuse in the vitreous, and they cannot permeate across the inner limiting membrane into the retina. Importantly, we demonstrate that AF4 is a useful tool for monitoring the aggregation properties of nanoparticles in the vitreous humor.

Ocular drug delivery is usually investigated using chemical analytical methods that require invasive sampling of ocular tissues, leading to high demand of laboratory animals, because many rabbits must be sacrificed at each time point of the kinetic curve [2]. Even though non-invasive ocular fluorophotometry has been used to quantitate intravitreal pharmacokinetics of soluble antibody [32], it has never before been applied to study intravitreal kinetics of nanomedicines. Here, we demonstrate that this method is quantitative and useful for this purpose. Continuous monitoring of the injected particles in the eye with fluorophotometry enables data generation with 4–8 times smaller number of animals as compared to the traditional invasive methods.

Drug delivery to the eye may be practiced also via other ocular routes of drug administration, such as suprachoroidal, sub-retinal, intra-scleral, and sub-tenon injections [2]. Polymersome technology may be applicable for many ocular injection routes, in which small injection needles and small unit size of injectable species are needed. Polymeric micelles and polymersomes may provide prolonged retention also at these injection sites.

It is evident that polymersomes and, to lesser extent, polymeric micelles are promising vehicles for intravitreal drug delivery. However, drug encapsulation and release are needed in effective drug treatment. We illustrate the potential of polymersomes as long-acting formulation by some kinetic calculations. We injected 320 µg of polymersomes that showed retention of several months in the vitreous. Let us assume retention of 3 months and drug loading of ≈1% results in a drug dose of 3 µg per injection. The average steady-state drug concentration (C_ss,av_) in the vitreous would be C_ss,av_ = J/CL, where J is the zero-order release rate (3 µg/3 months) and CL is the vitreal clearance for the released drug (typically about 0.05–0.5 mL/h) [2]. Then, steady state drug levels would be 2.8–28 ng/mL (e.g., for small molecule, mw. 280 Da, 10–100 nM). Even low levels of drug loading (1%) would be adequate for drugs that are active at 10^−7^ M. For first-order release kinetics, higher drug potency is required, but loading of 1% would work for drugs that require concentrations of 10^−9^–10^−8^ M for activity.

## 6. Conclusions

Intravitreally injected polymersomes and polymeric micelles were studied as potential intravitreal systems for drug delivery. We observed that the polymersomes retain in the vitreous for several months, reaching the retina and targeting the optic nerve head region. Polymersomes are a promising injectable formulation platform for long acting and targeted intravitreal drug delivery. Furthermore, a new fluorometric approach for non-invasive kinetic monitoring of nanomedicines was successfully introduced.

## Figures and Tables

**Table 1:** Diameters and zeta potentials of the polymeric particles.

Formulation (Polymer)	Size ± SD (nm)	Polydispersity Index (PDI)	Zeta Potential (mV)
Neutral polymersome (PPP)	95 ± 11	0.257	0.7
Cationic polymersome (pPPP)	115 ± 9	0.291	+13.2
Anionic polymersome (nPPP)	89 ± 19	0.297	−11.9
Polymeric micelle (p22)	31 ± 4	0.088	−2.7
Polymeric micelle (p42)	43 ± 6	0.054	−4.9

**Table 2:** Mobility parameters of polymersomes in the vitreous. D_v_ was experimentally derived from single particle analysis based on mean square displacement of the particles, and D_w_ was calculated using Stokes–Einstein equation.

Formulation (Polymer)	D_v_ (µm^2^/s)	D_w_ (µm^2^/s)	D_w_/Dv
Neutral polymersome (PPP)	0.40 ± 0.10	6.93	17.3
Cationic polymersome (pPPP)	0.33 ± 0.09	5.73	17.5
Anionic polymersome (nPPP)	0.48 ± 0.12	7.40	15.5

**Table 3:** Pharmacokinetic parameters obtained from fluorophotometry (*n* = 3) readings after intravitreal injections to the rabbits. Values for individual rabbits are shown. Neutral polymersomes and p22 polymer micelles were used in these experiments. Values of AUC (area under the curve), CL (clearance), and V_ss_ (steady state volume of distribution) are presented.

Formulation	Rabbit	AUC (h mg/mL)	Half-Life (Days)	CL (µL/h)	V_ss_ (mL)
Micelle	22	91.9	5.8	5.4	1.09
Micelle	20	124.7	9.2	4.0	1.28
Micelle	17	137.8	9.5	3.6	1.20
Micelle	15	122.7	4.3	4.1	0.60
Polymersome	21	36.8	11.4	8.7	3.42
Polymersome	19	193.7	32.7	1.7	1.87

## Data Availability

Data is contained within the article and supplementary material. The additional data presented in this study are available on request from the authors.

## Supplementary Materials

The following are available online at https://www.mdpi.com/article/10.3390/pharmaceutics13040445/s1, Scheme S1: Synthetic routes for the generation of block copolymers, Figure S1: 1H NMR (a) and SEC trace (b) of PEG22-PCLgTMC, Figure S2: 1H NMR (a) and SEC trace (b) of NH2-PEG42-PCLgTMC, Figure S3: 1H NMR (a) and SEC trace (b) of COOH-PEG42-PCLgTMC, Figure S4: 1H NMR (a) and SEC trace (b) of PEG22-PCLgTMC-BODIPY, Figure S5: Cell viability of HUVEC cells in the presence of polymeric micelles (p22, p42) and polymersomes (PPP, PPPP, NPPP) at different concentrations. The cells were treated with micelles or polymersomes for 24 h. Cell viability was evaluated with oxidation–reduction indicator resazurin (Alamar Blue^®^ Cell Viability Reagent). Data was normalized based on the viability of untreated cells (100%). Data are represented as mean ± SD (n = 3); Figure S6: Calibration curve for A) polymersomes (PPP) (fitted equation: Y = 0.524X + 25.58, R2 = 0.987) and B) polymeric micelles (p22) (fitted equation: Y = 0.117X + 40.41, R2 = 0.985), Figure S7: Axial distribution of polymeric micelles and polymersomes at the terminal time points. A, B, C and D are the fluorophotometric scans of polymeric micelles (p22). E and F show the scans of polymersomes. In the case of polymersomes, concentration gradients with higher levels near the retina are seen. The gradient is not significant in polymeric micelle data, Table S1: General method for the AFFF elution of polymersomes. The applied flow conditions were as follows: 0.7 mL min−1 detector flow, 1.50 mL min−1 focus flow and 0.20 mL min−1 injection flow, Table S2: General method for the AFFF elution of micelles. The flow conditions applied were the.following: 0.7 mL min−1 detector flow, 1.5 mL min−1 focus flow and 0.20 mL min−1 injection flow.

## Abbreviations

**Abbr.**	**Full Names**
ACN	acetonitrile
AF4	asymmetric flow field flow fractionation
BODIPY	boron-dipyrromethene;
CMC	critical micellar concentration
D_v_	diffusion coefficient in vitreous
DCM	dichloromethane
DLS	dynamic light scattering
DMEM	Dulbecco′s modified eagle′s medium
DPBS	Dulbecco’s phosphate buffered saline
ECGS	endothelial cell growth supplement
FBS	fetal bovine serum
GPC	gel permeation chromatography
MALS	multi-angle light scattering
MSD	mean square displacement
NMR	nuclear magnetic resonance
PEG	Poly(ethylene glycol)
PBS	phosphate buffered saline
PCL	poly(ε-caprolactone)
PTMC	poly(trimethylene carbonate)
ROP	ring-opening polymerization
RT	room temperature
τ	time delay for the calculated movement
tBOC	tert-butyloxycarbonyl
TFA	trifluoroacetic acid
THF	tetrahydrofuran
TMC	trimethylene carbonate
