# Supplementary material for: Intravitreal Polymeric Nanocarriers with Long Ocular Retention and Targeted Delivery to the Retina and Optic Nerve Head Region
Source: Pharmaceutics. 2021 Mar 26;13(4):445. doi: 10.3390/pharmaceutics13040445 (PMC8066548; doi:10.3390/pharmaceutics13040445)
Supplement: Supplementary file 1 [file pharmaceutics-13-00445-s001.pdf]

pharmaceutics

Supplementary materials: Intravitreal Polymeric Nanocarriers with Long Ocular Retention and Targeted Delivery to the Retina and Optic Nerve Head Region

Vijayabhaskarreddy Junnuthula, Amir Sadeghi Boroujeni, Shoupeng Cao, Shirin Tavakoli, Roxane Ridolfo, Elisa Toropainen, Marika Ruponen, Jan C. M. van Hest and Arto Urtti

Citation: Junnuthula, V.; Boroujeni, A.S.; Cao, S.; Tavakoli, S.; Ridolfo, R.; Toropainen, E.; Ruponen, M.; van Hest, J.; Urtti, A. Intravitreal Polymeric Nanocarriers with Long Ocular Retention and Targeted Delivery to the Retina and Optic Nerve Head Region. *Pharmaceutics* **2021**, *13*, 445. https:// doi.org/10.3390/pharmaceutics13040445Academic Editor: Armando Da Silva Cunha Júnior

Received: 6 March 2021
Accepted: 23 March 2021
Published: 26 March 2021

Publisher's Note: MDPI stays neutral with regard to jurisdictional claims in published maps and institutional affiliations.

Copyright: © 2021 by the authors. Submitted for possible open access publication under the terms and conditions of the Creative Commons Attribution (CC BY) license (http://creativecommons.org/licenses/by/4.0/).

1. Synthesis Protocols

The main features of the synthetic protocols have been presented in the Supplementary scheme.

1.1. Synthesis of poly(ethylene glycol)-b-(poly(ε-caprolactone)-g-poly(trimethylene carbonate)) (PEG-b-p(CL-g-TMC)) Block Copolymers

Monomethoxy/NHBoc/carboxyl-PEG-OH macroinitiator was weighed into a flame-dried round bottom flask along with ε-caprolactone (CL) and trimethylene carbonate (TMC). For all polymers, stoichiometric amounts of CL and TMC were added to the reaction mixture and dry toluene (ca. 50 mL) was then added to the flask. Toluene was subsequently evaporated in order to dry the contents. The dried reagents were then re-dissolved in dry dichloromethane (DCM) ([CL] + [TMC] = 0.5 M) and dry methane sulfonic acid (MSA) was added (1 equivalent vs PEG initiator) under argon. The reaction was continued at 30 °C overnight, until no sign of unreacted monomers was observed anymore. After the reaction was finished, the solution was concentrated and the copolymer precipitated into ice cold MeOH, followed by dissolution in dioxane and lyophilization to yield a wax at a yield of 80–90 %. Copolymer composition was ascertained from the NMR spectrum. The polymer chain length and the dispersity were determined by ^{1}H NMR and gel permeation chromatography (GPC).

1.2. Synthesis of NH_2-PEG-b-p(CL-g-TMC)

Boc-NH-PEG-b-p(CL-g-TMC) was dissolved in dichloromethane (DCM) and cooled on ice. While stirring, an equimolar amount of trifluoro acetic acid (TFA) (dropwise) was added, the ice bath was removed and the reaction was allowed to warm slowly to room temperature and it was stirred for 2 hours. Next, the solvent was removed by evaporation and then residual TFA was extracted using base washes, water and brine. The polymer was then precipitated in ether, dissolved in dioxane and freeze dried.

1.3. Synthesis of CO_2H-PEG-b-p(CL-g-TMC)

Monomethoxy/NHBoc/carboxyl-PEG-OH macroinitiator was weighed with CL and TMC in a flame-dried round bottom flask. For all polymers, stoichiometric amounts of CL and TMC were added to the reaction mixture and dry toluene (ca. 50 mL) was then added to the flask and evaporated in order to dry the contents. The dried reagents were then re-dissolved in dry DCM ([CL] + [TMC] = 0.5 M) and dry methane sulfonic acid (MSA) was added (1 equivalent vs initiator) under argon. The reaction was stored at 30 °C overnight, until all monomers had reacted. After the reaction was finished, the solution was concentrated and copolymer precipitated into ice cold methanol, followed by dissolution in dioxane and lyophilization to yield a wax at a yield of 80–90 %. Copolymer composition was ascertained from the NMR spectrum. The polymer chain length and the dispersity were determined by 1H NMR and GPC.

1.4. Synthesis of PEG-b-p(CL-g-TMC)-BODIPY

Approximately 370 mg of copolymer was dissolved in 5 mL of dry DCM into which *N,N′*-dicyclohexylcarbodiimide (DCC, 50 mg), N,N-diisopropylethylamine (DIPEA, 0.5 ml), 4-dimethylaminopyridine (DMAP, 10 mg) and 3-bodipy-propanoic acid (Bodipy-acid, 20 mg) were added. The solution was stirred at room temperature for 48 hours before the fluorescent product was dialyzed extensively in a 3.5 kDa molecular weight cut-off membrane into DCM and dioxane prior to lyophilization.

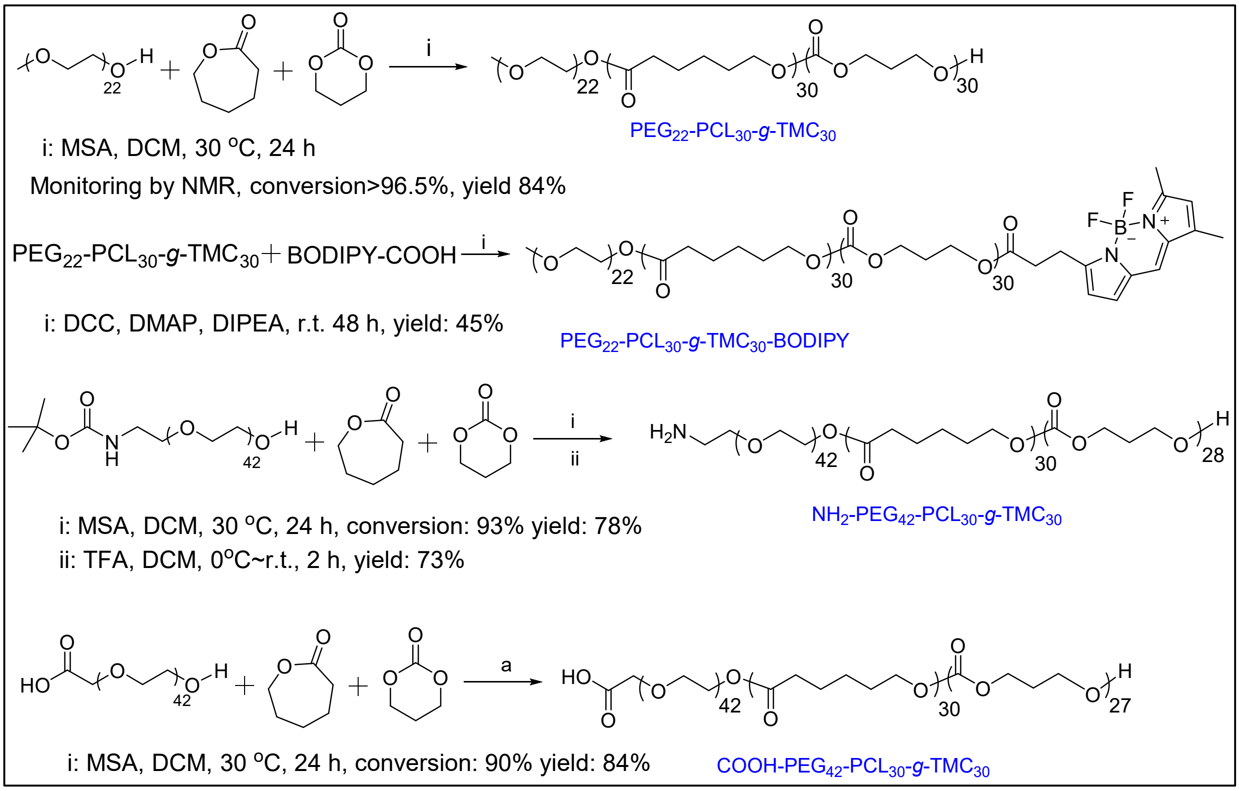

Scheme S1. Synthetic routes for the generation of block copolymers.

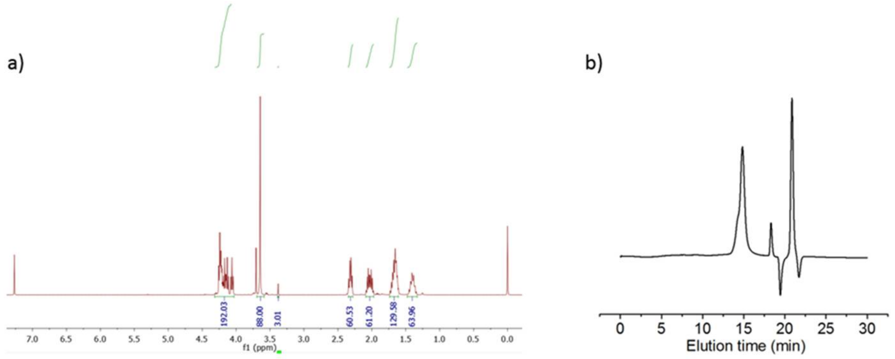

Figure S1. ^{1}H NMR (**a**) and SEC trace (**b**) of PEG22-PCLgTMC.

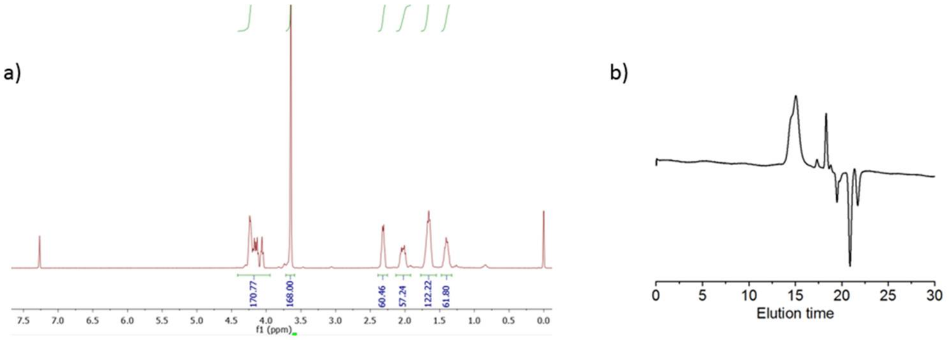

Figure S2. ^{1}H NMR (**a**) and SEC trace (**b**) of NH_2-PEG_{42}-PCLgTMC.

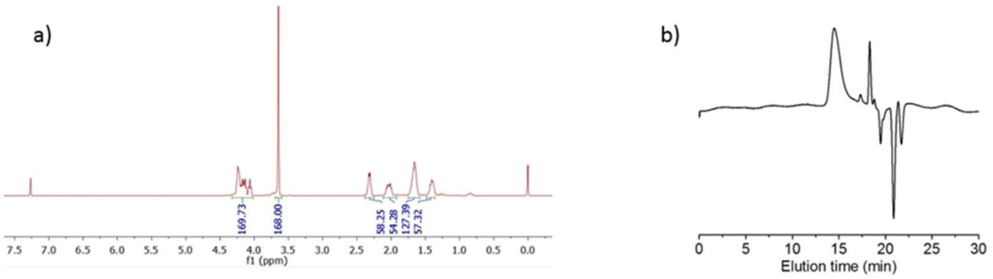

Figure S3. ^{1}H NMR (**a**) and SEC trace (**b**) of COOH-PEG_{42}-PCLgTMC.

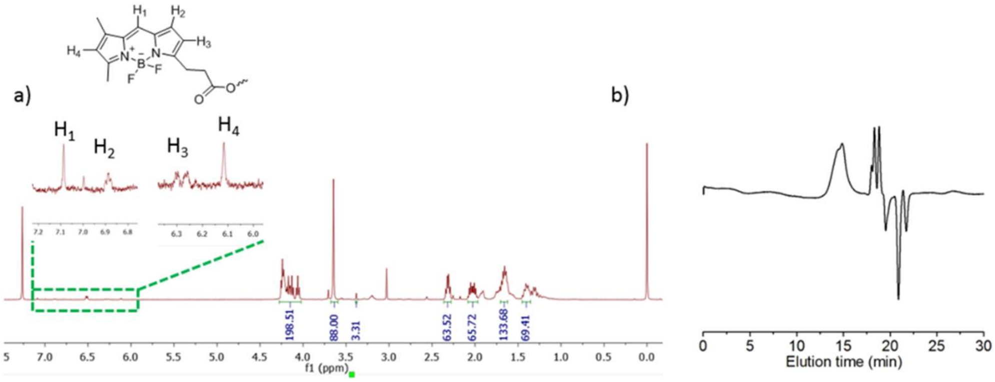

Figure S4. ^{1}H NMR (**a**) and SEC trace (**b**) of PEG_{22}-PCLgTMC-BODIPY.

2. AFFF Experiments

Table S1. General method for the AFFF elution of polymersomes. The applied flow conditions were as follows: 0.7 mL min^{-1} detector flow, 1.50 mL min^{-1} focus flow and 0.20 mL min^{-1} injection flow.

Start Time (min)	End Time (min)	Mode	Cross Flow Start (mL/min)	Cross Flow End (mL/min)
0	1	Elution	0.50	0.50
1	2	Focus	-	-
2	3	Focus + inject	-	-
3	4	Focus	-	-
4	19	Elution	0.50	0.50
19	22	Elution	0.50	0.00
22	28	Elution	0.00	0.00
28	29	Elution + inject	0.00	0.00

29	30	Elution	0.00	0.00

Table S2. General method for the AFFF elution of micelles. The flow conditions applied were the following: 0.7 mL min^{-1} detector flow, 1.5 mL min^{-1} focus flow and 0.20 mL min^{-1} injection flow.

Start Time (min)	End Time (min)	Mode	Cross Flow Start (mL/min)	Cross Flow End (mL/min)
0	1	Elution	0.40	0.40
1	2	Focus	-	-
2	3	Focus + inject	-	-
3	4	Focus	-	-
4	17	Elution	0.40	0.40
17	18	Elution	0.40	0.20
18	31	Elution	0.2	0.2
31	33	Elution	0.20	0.00
33	34	Elution + inject	0.00	0.00
34	35	Elution	0.00	0.00

3. Cellular Toxicity

HUVEC cells and culture medium were purchased from PromoCell GmbH (Heidelberg, Germany). All other cell culture media and supplements were purchased from Gibco (Invitrogen, CA, USA) except heparin and endothelial cell growth supplement (ECGS) that were bought from Sigma-Aldrich (St. Louis, MO, USA).

Cell viability in the presence of the polymersomes and polymeric micelles was studied using human umbilical vein endothelial cells (HUVEC, P6–P9). HUVEC cells were cultured in Ham's F-12K medium with heparin (0.1 mg/ml), endothelial cell growth supplement (ECGS) (0.05 mg/ml), 10% FBS, 1% l-glutamine, and 1% penicillin–streptomycin. The cell viability was studied using an Alamar Blue assay (Life Technologies GmbH, Darmstadt, Germany). The HUVEC cells (5000 per well) were seeded on ~~a~~ tissue culture treated Black Isoplate-96 plates and cultured for 48 h in an incubator (37 °C, 5% CO_2). The nanocarriers (25 µl) were added at different concentrations to the cell culture medium. The cells were incubated for 24 h in an incubator and then washed with cell culture grade PBS. Then, the cell viability reagent Alamar Blue was added to the cells at the ratio of 10:1 (serum free medium:Alamar Blue). The cells were incubated for 3 h at 37 °C and their viability was analyzed with a 96-well plate reader (Varioscan, Thermo Fisher Scientific) using excitation and emission wavelengths of 570 and 585 nm, respectively, and a bandwidth of 5 nm. Control cells without polymersome exposure were used as controls.

The Alamar Blue cell viability assay showed that the polymeric micelles and polymersomes were not toxic to the HUVEC cells (Figure S5) even at high concentrations.

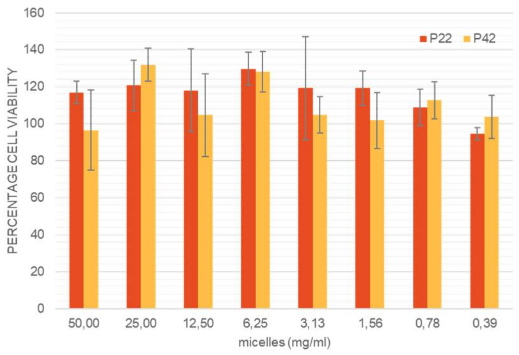

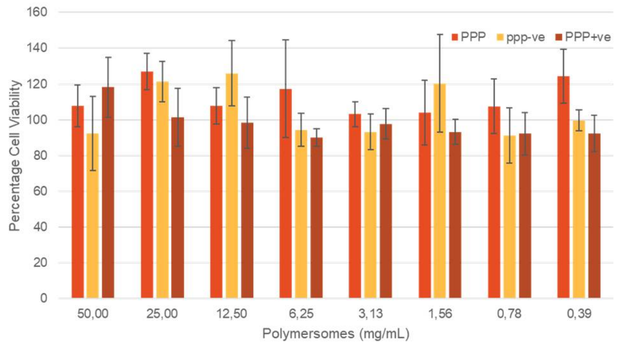

Figure S5. Cell viability of HUVEC cells in the presence of polymeric micelles (p22, p42) and polymersomes (PPP, PPPP, NPPP) at different concentrations. The cells were treated with micelles or polymersomes for 24 hours. Cell viability was evaluated with oxidation–reduction indicator resazurin (Alamar Blue® Cell Viability Reagent). Data was normalized based on the viability of untreated cells (100%). Data are represented as mean ± SD (n = 3).

4. In Vivo Fluorophotometry

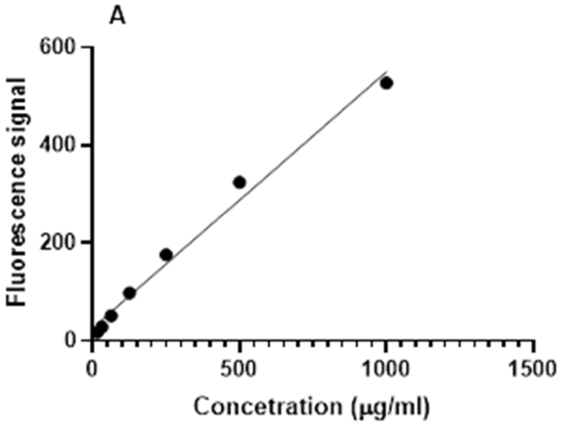

Figure S6. Calibration curve for **A**) polymersomes (PPP) (fitted equation: Y = 0.524X + 25.58, R^2 = 0.987) and **B**) polymeric micelles (p22) (fitted equation: Y = 0.117X + 40.41, R^2 = 0.985).

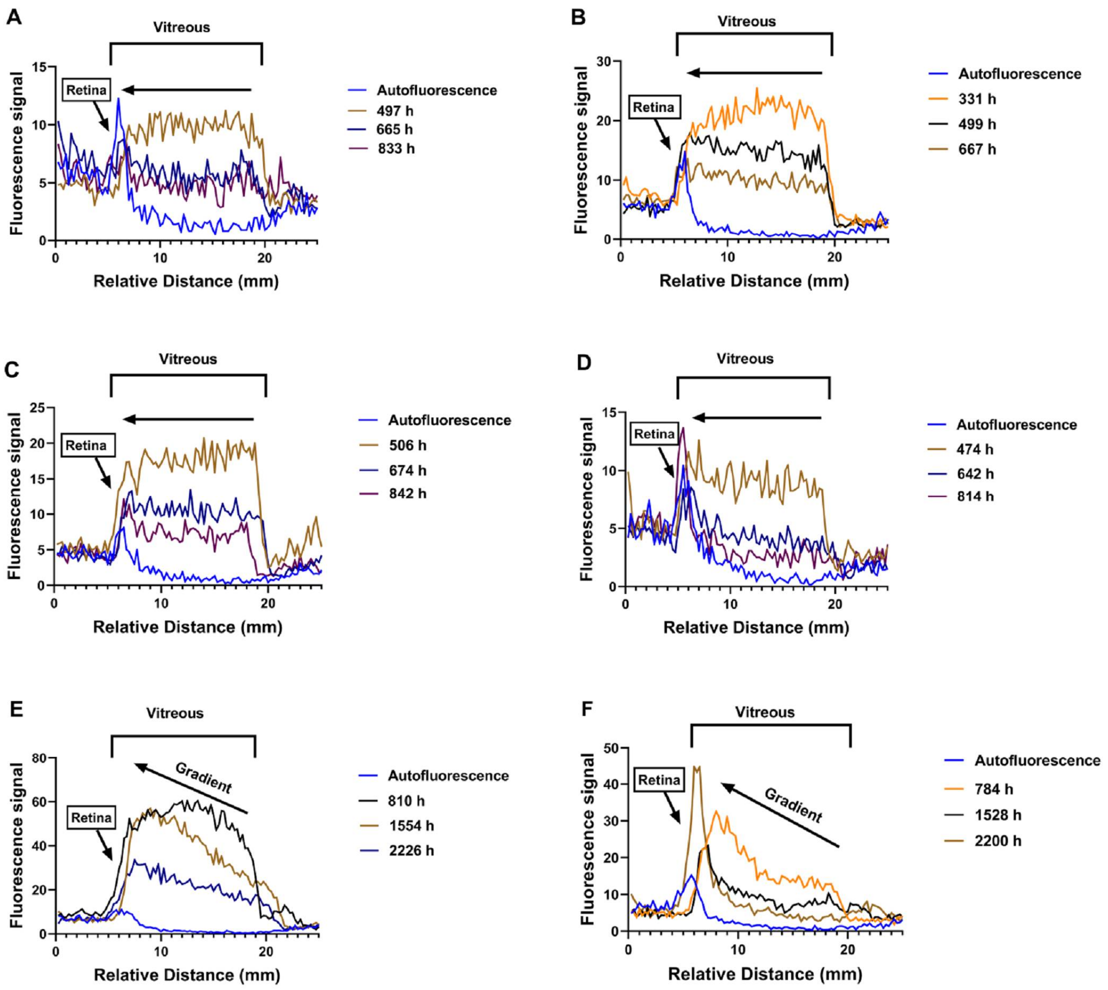

Figure S7. Axial distribution of polymeric micelles and polymersomes at the terminal time points. **A**, **B**, **C** and **D** are the fluorophotometric scans of polymeric micelles (p22). **E** and **F** show the scans of polymersomes. In the case of polymersomes, concentration gradients with higher levels near the retina are seen. The gradient is not significant in polymeric micelle data.
